# Outbreak response activities conducted by public health programs in healthcare facilities nationwide, August 2019–July 2020

**DOI:** 10.1017/ash.2022.82

**Published:** 2022-05-16

**Authors:** Nijika Shrivastwa, Lucas Ochoa, Maroya Walters, Kiran Perkins, Joseph Perz, Jennifer C. Hunter

## Abstract

**Background:** Rapid response is critical to control healthcare-associated infection (HAI) and antibiotic resistance threats within healthcare facilities to prevent illness among patients, residents, and healthcare personnel. Through this analysis, we aimed to quantify public health response activities, by healthcare setting type, for (1) novel and targeted multidrug-resistant organisms or mechanisms (MDROs), (2) SARS-CoV-2, and (3) other possible outbreaks. **Method:** We reviewed response activity data submitted by US state, territorial, and local health department HAI/AR programs to the CDC as part of funding requirements. We performed descriptive analyses of response activities conducted during the funding reporting period (August 2019–July 2020). SARS-CoV-2 response activities were reported from January through July 2020. Data were analyzed by response category (novel or targeted MDRO, SARS-CoV-2, other HAI/AR responses), and healthcare setting type. **Results:** During August 2019–July 2020, 57 HAI/AR Programs (50 state, 1 territorial, 5 local health departments, and District of Columbia) reported 18,306 public health responses involving healthcare facilities. These data included 3,860 responses to 1 or more cases of novel or targeted MDROs, 13,992 responses to SARS-CoV-2 outbreaks (beginning in January 2020), and 454 responses to other possible outbreaks. Novel and targeted MDRO responses most frequently occurred in acute-care hospitals (ACHs, 64.5%), skilled nursing facilities (SNFs, 24.5%), and long-term acute-care hospitals (LTACHs, 5.8%). SARS-CoV-2 responses most frequently occurred in SNFs (55%), and assisted living facilities (24%). Other HAI/AR responses most frequently occurred in ACH (50%), SNF (28.4%), and outpatient settings (19.6%). Of the “other” HAI/AR responses, 76% were responses to cases, clusters, or outbreaks, and 23.8% were responses to serious infection control breaches including device and instrument reprocessing, injection safety, and other deficient practices. **Conclusions:** During the study period, public health programs performed a high volume of HAI/AR response activities largely focused on SARS-CoV-2 in nursing homes and assisted living facilities. Other important response activities occurred across a range of other healthcare settings, including responses to novel and targeted MDROs, HAI outbreaks, and serious infection control breaches. Whereas SARS-CoV-2 response activities largely centered in long-term care settings, MDRO and other HAI/AR responses occurred mostly in acute-care settings. These data demonstrate the importance of building and sustaining public health response capacity for a broad array of healthcare settings, pathogens, and patient populations to meet the range of current and emerging HAI/AR threats.

**Funding:** None

**Disclosures:** None

